# Campylobacteriosis, Eastern Townships, Québec

**DOI:** 10.3201/eid1010.040228

**Published:** 2004-10

**Authors:** Sophie Michaud, Suzanne Ménard, Robert D. Arbeit

**Affiliations:** * Faculté de Médecine de l'Université de Sherbrooke, Québec, Canada;; † Régie Régionale de la Santé et des Services Sociaux de l'Estrie, Québec, Canada;; ‡ Boston University School of Medicine, Boston, Massachusetts, USA

**Keywords:** Campylobacter, case-control studies, risk factors, epidemiology, chickens, dispatch

## Abstract

Independent risk factors for campylobacteriosis (eating raw, rare, or undercooked poultry; consuming raw milk or raw milk products; and eating chicken or turkey in a commercial establishment) account for <50% of cases in Québec. Substantial regional and seasonal variations in campylobacteriosis were not correlated with campylobacter in chickens and suggested environmental sources of infection, such as drinking water.

Published case-control studies provide conflicting results regarding the risk factors for sporadic campylobacteriosis. Poultry is commonly considered the principal source, and in some studies, was implicated in 50% to 70% of endemic cases (*1**,**2*). *Campylobacter* have been frequently cultured from poultry during processing (47%–82%) and retail distribution (23%–62%) (*3**–**6*). However, some studies observed no significant risk associated with eating chicken (*7**,**8*); in other studies, this factor was actually protective (*9**,**10*). We describe a prospective case-control study of domestically acquired *Campylobacter* infections combined with a prevalence study of *Campylobacter* spp. in whole retail chickens purchased in the Eastern Townships, Québec.

## The Study

The Eastern Townships comprise seven counties and total ≈300,000 inhabitants. Hospital microbiology laboratories routinely report all *Campylobacter* enteritis cases to the regional public health department. All the laboratories in the study region, except in Granit County, routinely evaluated stool specimens for *Campylobacter* by using comparable standard methods for isolation and identification (Karmali or Skirrow media incubated for 72 h at 42°C in a microaerobic atmosphere). Granit County's laboratory sent stool specimens for *Campylobacter* culture to our hospital microbiology laboratory on special medical request only. Incidence rates of campylobacteriosis in the Eastern Townships and Québec Province were calculated with demographic and reportable diseases data from provincial registers.

All cases reported from July 1, 2000, through September 30, 2001, were eligible. Case-patients were excluded if the infection was acquired outside Québec (i.e., travel abroad during the 10-day period before the onset of symptoms) or if the interval between the onset of symptoms and reporting was >6 weeks. All investigations were conducted within 2 weeks of reporting. For participants with infections reported on multiple occasions during the study period, the first episode of infection was considered. The median interval from the onset of symptoms to the interview of the cases was 13 days (range 5–56 days; 90th percentile, 23 days).

Each case was matched for sex and age group (<1, 1–4, 5–14, 15–34, 35–64, and >65 years) to two controls living in the Eastern Townships, who were identified through random digit dialing. Patients and controls were interviewed by telephone with a structured questionnaire to capture demographic and clinical data, travel history, food history, water consumption, recreational water activity, animal contacts, and other illness during the 10 days before the onset of symptoms. Controls had to be interviewed within 3 weeks of the patient and were excluded if they could not be reached after three telephone calls; had fever, abdominal pain, nausea, vomiting, diarrhea, or bloody stools; traveled abroad during the 10-day period before the patient's onset of symptoms; or refused to participate. Controls did not have stool samples tested for *Campylobacter*. A surrogate parent was interviewed when the patient or control was a child <14 years of age. The interviewers were not blinded to the patient or control status of study participants.

Risk factors for campylobacteriosis were evaluated by conditional logistic regression for matched data adjusted for the county of residency. All risk factors with p < 0.05 by univariate analysis were included in a multivariate, conditional, logistic regression, stepwise selection model for matched data. All statistical analyses were performed using SAS version 6.1 (SAS, Cary, NC).

During the study, four fresh, eviscerated whole chickens were bought weekly in different counties (one chicken per store); for each county, the number of chickens sampled monthly was proportional to the population. Retail chickens sold in the Eastern Townships are produced by multiple companies based elsewhere in Québec Province.

The chickens were stored at 4°C overnight and washed vigorously with 250 mL of nutrient broth. The broth was filtered through cheesecloth and centrifuged at 16,300 x *g* for 15 min. The sediment was suspended in 5 mL of brucella broth; 100 mL of Park and Sanders' selective enrichment broth with 0.5 mL of Supplement A (0.2% vancomycin and 0.2% trimethoprim lactate) and 5 mL of Supplement B (0.064% sodium cefoperazone in brucella broth) (*11*) were added to the suspension, gently mixed, and incubated under microaerobic atmosphere at 37°C for 4 h, then at 42°C for 48 h. Three loopfuls (0.05 mL) of the suspension were plated on Karmali agar and incubated at 42°C for 48 h under microaerobic conditions. Isolates of *Campylobacter* were identified to the species level by routine phenotypic methods.

From July 2000 through October 2001, a total of 201 cases of campylobacteriosis were reported, of which 43 were excluded: 18 patients acquired their infection outside Québec, 18 resided outside the Eastern Townships, 6 could not be interviewed within 6 weeks after the onset of symptoms, and 1 patient declined to participate. All but two patients were matched to two controls each; consequently, the final dataset comprised 158 cases and 314 controls. Cases and controls were well-distributed across the seven counties, except in Val St-François, which represented 15% of cases and 7% of controls (data not shown).

**Figure 1:**
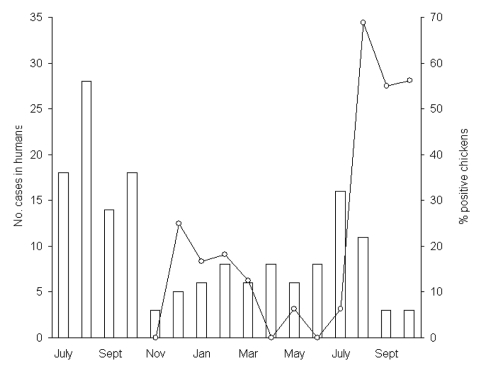
Monthly distribution of the number of sporadic cases of *Campylobacter* infections in humans from July 2000 to October 2001 (columns) and of the prevalence of *Campylobacter* in whole retail chickens from November 2000 to October 2001 (line graph).

**Figure 2:**
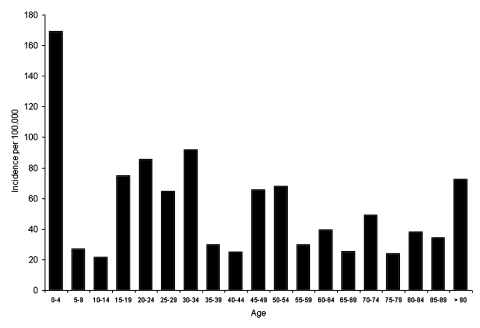
Distribution of the incidence rates of *Campylobacter* infection by age in the Eastern Townships.

During the study period, the mean crude incidence of campylobacteriosis was 63.1/100,000 in the Eastern Townships, compared to 44.5/100,000 in the remainder of Québec Province (p < 0.0001). Most cases occurred during July, August, and September (Figure 1). The median age of the case-patients was 31 years (range 11 days to 91 years). The incidence of campylobacteriosis varied considerably by age (Figure 2), with the highest rates among children 0–4 years of age (169.2/100,000) and young adults 15–34 years of age (mean = 79.4/100,000). Overall, 64 (40.5%) participants were female.

The rates varied from 38.3/100,000 in Memphrémagog to 113.5/100,000 in Asbestos (excluding Granit, where case ascertainment was different); these interregional differences persisted after stratification for age (Table 1). The risk of campylobacteriosis was 2.4-fold higher in Asbestos (p = 0.0001) and 1.3-fold higher in Val St-François (p = 0.04) than elsewhere in the Eastern Townships.

**Table 1:** Incidence rates of campylobacteriosis in each county in the Eastern Townships with crude and age-stratified relative risk compared with incidence rates in the other counties in the region^a^

County	No. of cases	Total population	Incidence rate per 100,000	Crude RR	RR stratified for age^b^	p value^c^
Asbestos	17	14,975	113.5	2.23	2.37	0.0001
Val St-François	23	28,809	79.8	1.48	1.33	0.04
Sherbrooke	85	143,792	59.1	1.09	1.14	NS
Coaticook	9	16,444	54.7	0.97	0.85	NS
Memphrémagog	16	41,785	38.3	0.64	0.79	NS
Haut St-François	10	22,358	44.7	0.78	0.74	NS
Granit	4	21,905	ND^d^	ND^d^	ND^d^	ND^d^

^a^NS, not significant; ND, not done. ^b^The relative risk (RR) represents the incidence rate of campylobacteriosis in one county compared to the incidence rate in the other counties taken as a whole, before (crude RR) and after stratification for age (stratified RR). ^c^The p values apply to the stratified relative risks. ^d^Rates not calculated for County of Granit because of different case ascertainment process used there; see text for details.

Among 41 exposure factors evaluated by univariate conditional logistic regression, four achieved p values < 0.01 (Table 2). Two were associated with poultry: eating raw, rare, or undercooked poultry (p = 0.003) and eating turkey or chicken in a restaurant, a fast food establishment, or a buffet (p = 0.004). Two were associated with other exposures: consuming raw milk or raw milk products (p = 0.0001) and professional exposure to animals or a contact with farm or zoo animals (p = 0.0003). No other activity related to consuming or handling poultry appeared related to infection (Table 2).

**Table 2:** Exposure factors significantly associated with campylobacteriosis^a^ and other factors relating to consuming and handling poultry^b,c^

Factor	Case-patients	Controls	OR	95% CI
Eating raw, rare or undercooked poultry	13/154	7/310	4.51	1.67–12.14
Consuming raw milk or raw milk products	33/153	25/310	3.12	1.78–5.48
Professional exposure to animals or contact with farm or zoo animals	39/158	36/312	2.53	1.44–4.13
Eating turkey or chicken in a restaurant, a fast food restaurant, or a buffet	57/140	77/289	1.89	1.23–2.90
Eating smoked turkey or chicken				
In a restaurant, a fast food restaurant, or a buffet	5/156	6/309	1.67	0.50–5.57
At home	42/153	93/310	0.90	0.58–1.38
Eating poultry cooked in fondue	5/156	7/312	1.49	0.46–4.79
Eating microwaved poultry	2/158	3/309	1.36	0.22–8.26
Eating barbecued poultry	34/157	66/310	1.02	0.64–1.64
Handling raw poultry	78/153	160/314	0.97	0.66–1.44
Eating microwaved chicken croquettes	5/157	11/308	0.92	0.31–2.72
Using the same plate to carry raw meat or poultry and to take it back once cooked	38/156	66/302	0.78	0.48–1.29
Eating turkey or chicken at home	128/140	274/289	0.58	0.26–1.27
Eating ground turkey or chicken	3/158	12/314	0.50	0.14–1.79

^a^By univariate conditional logistic regression for matched data adjusted for the county of residency. ^b^Not associated with campylobacteriosis. ^c^OR, odds ratio; CI, confidence interval.

Conditional multivariate analysis adjusted for the county of residency resolved only three independent risk factors: raw, rare, or undercooked poultry (odds ratio [OR] 5.00, 95% confidence interval [CI] 1.79–13.98, p = 0.002), raw milk or raw milk products (OR 3.67, 95% CI 1.95–6.90, p = 0.0001), and turkey or chicken eaten in a restaurant, a fast food or a buffet (OR 1.96, 95% CI 1.24–3.11, p = 0.004). These factors accounted for 8%, 18%, and 20% of cases, respectively.

A total of 177 chickens from 58 different food stores were cultured (median per month, 16; range 8–20). *Campylobacter* spp. were cultured from 41 (23%) (37 *C. jejuni*; 4 *C. coli*). The prevalence of *Campylobacter* was low from November 2000 to July 2001 inclusively, with 0–2 positive chickens (0%–25%) per month (Figure 1) but increased sharply in August, September, and October 2001, with rates reaching 69%, 55%, and 56%, respectively. The number of locally acquired *Campylobacter* enteritis in humans peaked at 16 cases in July 2001 (i.e., 1 month before the peak of chicken contamination) and then decreased to 11, 3, and 3 cases in August, September, and October 2001, respectively. Further, we analyzed data for each county separately and found no geographic correlation between campylobacteriosis in humans and *Campylobacter* in chickens (p = 0.42). Thus, although chicken consumption is an important risk factor for *Campylobacter* enteritis, it does not explain either the seasonal or regional variations in the incidence of sporadic cases of campylobacteriosis in humans.

## Conclusions

Exposures to poultry account for fewer than half the episodes of sporadic *Campylobacter* infection. Substantial seasonal and interregional variations suggest environmental sources of infection. In the univariate analysis, drinking tap water at home or at work tended to be associated with an increased risk for infection (OR 1.90, p = 0.03), and in a subanalysis of cases in Asbestos County, which had the highest incidence, drinking tap water from a deep well at home was the only risk factor identified (53% of cases compared to 23% of controls; OR 3.83, p = 0.06 by univariate analysis and OR 3.96, p = 0.06 after adjusting for age group and sex). A recent case-control study (*12*) identified drinking water that was not disinfected as an independent risk factor for campylobacteriosis, with an etiologic fraction of 26%. These results are consistent with the hypothesis that the waterborne route of infection may be the common underlying pathway linking infection in humans, poultry, other domestic animals, and wild birds.

In waterborne outbreaks associated with *Campylobacter*, fecal contamination of the drinking water source has been traced to runoff of surface water after rain or to leakage from a sewage line into an adjacent drinking water pipe (*13**–**15*). Since a few hundred viable organisms represent an infectious dose, even apparently low levels of contamination could result in infection. The true importance of drinking water as a source of sporadic infection in humans may have been underestimated in the past and should be investigated in future studies.
